# Epitranscriptomic investigation of myopia-associated RNA editing in the retina

**DOI:** 10.3389/fnins.2023.1220114

**Published:** 2023-06-28

**Authors:** Xu-Bin Pan, Yu-Shan He, Zijing Lu, Hao-Ran Pan, Zhi-Yuan Wei, Yun-Yun Jin, Jihong Wang, Jian-Huan Chen

**Affiliations:** ^1^Department of Ophthalmology, Affiliated Hospital of Jiangnan University, Wuxi, China; ^2^Laboratory of Genomic and Precision Medicine, Wuxi School of Medicine, Jiangnan University, Wuxi, Jiangsu, China; ^3^Joint Primate Research Center for Chronic Diseases, Institute of Zoology of Guangdong Academy of Science, Jiangnan University, Wuxi, Jiangsu, China; ^4^Jiangnan University Brain Institute, Wuxi, Jiangsu, China

**Keywords:** A-to-I RNA editing, the retina, myopia, form deprivation, epitranscriptome

## Abstract

Myopia is one of the most common causes of vision loss globally and is significantly affected by epigenetics. Adenosine-to-inosine (A-to-I RNA) editing is an epigenetic process involved in neurological disorders, yet its role in myopia remains undetermined. We performed a transcriptome-wide analysis of A-to-I RNA editing in the retina of form-deprivation myopia mice. Our study identified 91 A-to-I RNA editing sites in 84 genes associated with myopia. Notably, at least 27 (32.1%) of these genes with myopia-associated RNA editing showed existing evidence to be associated with myopia or related ocular phenotypes in humans or animal models, such as very low-density lipoprotein receptor (*Vldlr*) in retinal neovascularization and hypoxia-induced factor 1 alpha (*Hif1a*). Moreover, functional enrichment showed that RNA editing enriched in FDM was primarily involved in response to fungicides, a potentially druggable process for myopia prevention, and epigenetic regulation. In contrast, RNA editing enriched in controls was mostly involved in post-embryonic eye morphogenesis. Our results demonstrate altered A-to-I RNA editing associated with myopia in an experimental mouse model and warrant further study on its role in myopia development.

## Introduction

Myopia is among the most common refractive errors globally (Holden et al., 2016). As the leading cause of visual impairment in children, its incidence is increasing rapidly, especially in Asia. Myopia is prevalent among 80–90% of young adults in Asian urban areas, and of these cases, 10–20% are considered high myopia (Chua and Foster, 2020). According to the World Health Organization (WHO), it is estimated that by 2050, there will be 4,758 million people with myopia and 938 million people with high myopia globally, with an increase from 22% in 2000 to 52% by 2050 (Holden et al., 2016).

Myopia is caused by a complex interplay between common genetic and environmental factors, yet the exact mechanisms are not fully understood (Tedja et al., 2019). Linkage, candidate genes, and genome-wide association studies (GWAS) have identified genetic loci associated with myopia and refractive errors (Hysi et al., 2020). On the other hand, environmental factors, such as near work and outdoor exposure, could contribute to myopia development (Morgan et al., 2018). Moreover, myopia could also be influenced by interactions between genes and environmental factors, such as education (Clark et al., 2022).

Understanding gene–environment interactions in myopia requires understanding the crucial role that epigenetics has in the process. Seow et al. (2019) reported DNA methylation changes significantly associated with early-onset myopia risk by analyzing genome-wide DNA methylation profiles of umbilical cord samples from a cohort of Singaporean infants. In addition, the PCDHA gene cluster showed decreased DNA methylation levels in young Polish children with early-onset high myopia (Swierkowska et al., 2022). Nevertheless, there are only a few reported epigenetic findings on myopia, and it is necessary to understand other epigenetic processes in the disease aside from DNA methylation. RNA editing is an epigenetic process that post-transcriptionally alters the RNA sequence. Adenosine-to-inosine (A-to-I) RNA editing is the predominant type of RNA editing in vertebrates (Zinshteyn and Nishikura, 2009). Dysregulation of RNA editing has been associated with the pathogenesis of various neurological and neurodevelopmental disorders (Yang et al., 2021a), while its role in myopia remains uninvestigated.

Form deprivation myopia (FDM) is experimental myopia induced by blocking the regular visual input to one eye with a diffuser or an opaque lens. FDM animal models are used to study the mechanisms of human myopia and the role of epigenetic factors in its development. FDM animal studies have identified several epigenetic markers and pathways associated with myopia and its progression. A recent study on FDM mice suggested that DNA methylation of the *Col1a1* promoter/exon 1 might reduce scleral collagen production and lead to myopia development (Zhou et al., 2012).

In the current study, we performed an epitranscriptome analysis to investigate RNA editing in the retina of a mouse model of formal deprivation myopia. Our findings show altered A-to-I RNA editing in an experimental myopia mouse model and warrant further study on the role of A-to-I RNA editing in the molecular mechanism of myopia.

## Materials and methods

### RNA-seq dataset

FASTQ files of RNA-Seq raw reads were retrieved from the Gene Expression Omnibus (GEO).^1^ The dataset (PRJNA832969) contains 12 retinal samples of male C57BL/6 J mice from six FDM and six fellow controls, and each sample contains three retinas (Li et al., 2022). According to the original study, FDM was induced in the male C57BL/6 J mice at three weeks of age. After four weeks of FDM induction, the mouse retinas were collected, from which the total RNA was extracted and subjected to library construction using the ribosomal RNA depletion method. The library was sequenced with the HiSeq X Ten platform.

### Read mapping and processing

The sequencing data were processed as previously described (Tao et al., 2021). Sequencing read quality was analyzed using FASTQC. Clean data were aligned to the mouse genome sequence (UCSC mm10) using RNA STAR (version 2.7.0e) (Dobin and Gingeras, 2015). Multiple-mapped and duplicated reads were removed using SAMtools (version 1.9) (Li et al., 2009). Base quality scores were recalibrated using GATK (version 4.1.3) (Van der Auwera et al., 2013).

### Variant-calling, annotation, and filtering

Variants were called to identify single nucleotide variations (SNVs) by using VarScan (version 2.4.3) (Koboldt et al., 2012) and filtered using a bioinformatic pipeline as described in our previous study (Tao et al., 2021). In brief, SNVs were called using parameters base quality ≥25, total sequencing depth ≥ 10, alternative allele depth ≥ 2, and alternative allele frequency (AAF) ≥ 1%, and annotated using the Ensembl Variant Effect Predictor (VEP) (McLaren et al., 2016). As A-to-I editing was recognized as A-to-G in cDNA synthesis, A-to-G SNVs in the coding strand of genes, except for those annotated in the REDIportal V2.0 database (Mansi et al., 2021), were further filtered out and removed if located in the mitochondria, homopolymer runs ≥ five nucleotides (nt) or simple repeats, ≤ six nt from splice junctions, one nt from insertion-deletions, 4% to read ends, annotated in the dbSNP database Build 142, > 90% of samples with an AAF equal to 100% or between 40 and 60%, or with editing levels ≥1% in no more than one sample. The remaining high-confidence A-to-G SNVs were eventually included in subsequent data analysis.

### Protein–protein interactive network and gene function enrichment analysis

Protein–protein interactive (PPT) network construction and gene function enrichment analysis were conducted using the STRING database (Szklarczyk et al., 2021). Items with a false discovery rate (FDR) < 0.05 were considered significant.

### Statistical analysis

The intergroup editing levels of RNA editing sites were compared using the generalized linear model (GLM) and likelihood ratio test (LRT) to calculate the empirical GLM *p*-values. For RNA editing sites with empirical GLM *p* < 0.05, *Fisher*’s exact test was performed to calculate Fisher’s *P* for intergroup comparisons of the total counts of the reference and alternative alleles between the FDM and control groups. The Benjamini-Hochberg method for FDR calculation was used to adjust empirical *p*-values for multiple comparisons. Differentially edited RNA editing sites were defined as GLM FDR < 0.2 or *Fisher*’s exact test FDR < 0.2. Principal component analysis (PCA) was performed using R (version 3.6.3).

## Results

### Retinal A-to-I RNA editing identified in the epitranscriptome

**Figure 1:**
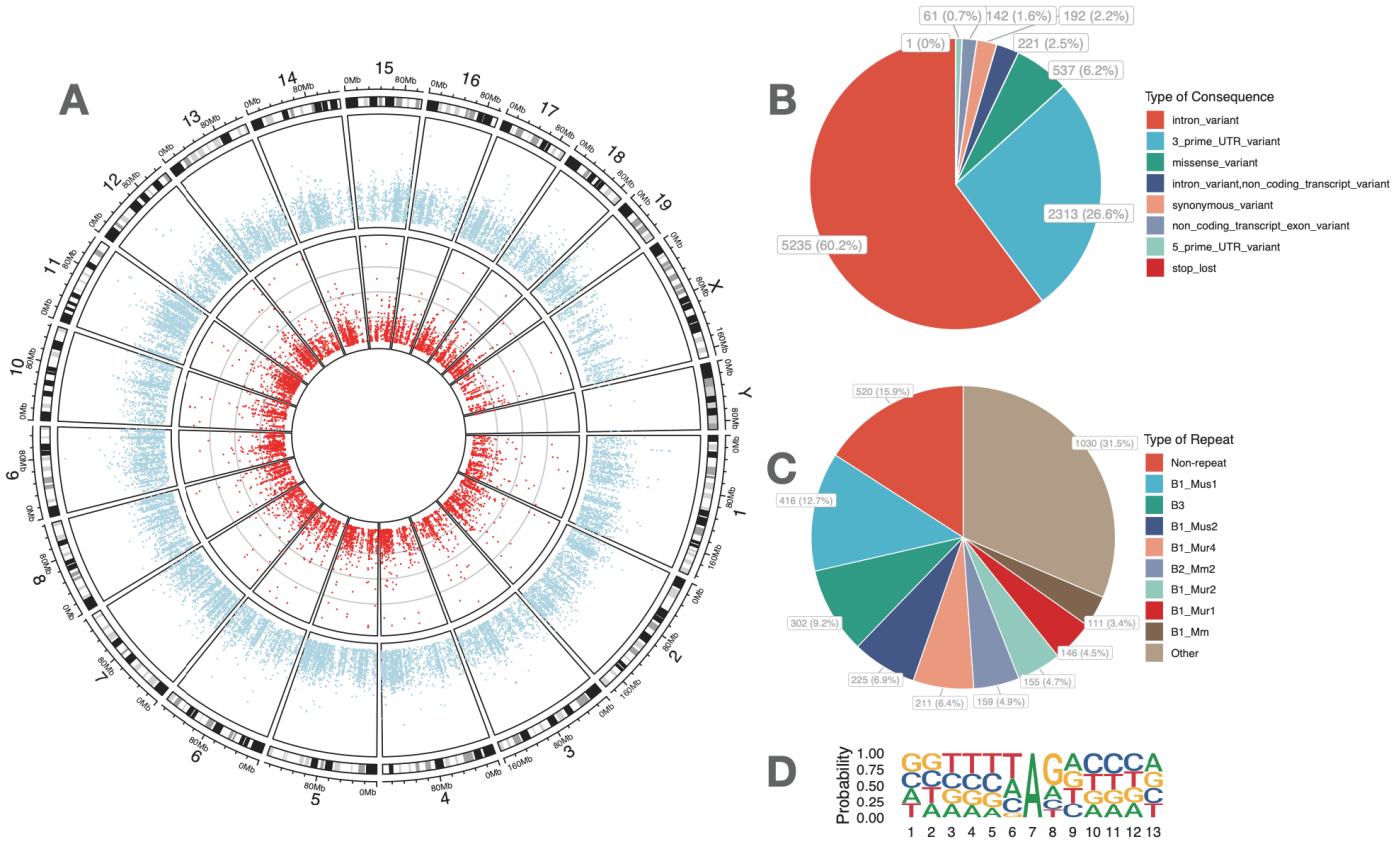
High-confidence A-to-I RNA editing sites were identified from the retinal transcriptome of adult mice. **(A)** Dots in red denote the average RNA editing level of A-to-I RNA editing sites, and blue dots denote the average RNA expression levels of genes. **(C)** Functional categories of these A-to-I RNA editing sites. **(D)** The distribution of repetitive elements overlapping with the A-to-I RNA editing sites. **(B)** The motif of sequence context surrounding the A-to-I RNA editing sites. Six nucleotides upstream and downstream of the editing sites are shown.

In all, 8,702 high-confidence A-to-I RNA editing events were observed in the RNA of 2,772 genes from all mouse retina samples (Figure 1A; Supplementary Table S1). These included 5,235 (60.2%) protein-coding intronic sites, 2,313 (26.6%) 3′-untranslated region (UTRs) sites, 221 (2.5%) non-coding transcript intronic sites, 537 (6.2%) missense sites, 192 (2.2%) synonymous sites, 142 (1.6%) non-coding transcript exonic sites, 61 (0.7%) 5′-UTR sites, and one (0.0001%) stop-lost site (Figure 1B). The repeats that most overlapped with these sites belonged to the B1 family (Figure 1C). Motif analysis of flanking sequence showed that G was suppressed at one base upstream but preferred at one base downstream of the editing sites (Figure 1D).

### Myopia-associated retinal A-to-I RNA editing

**Figure 2:**
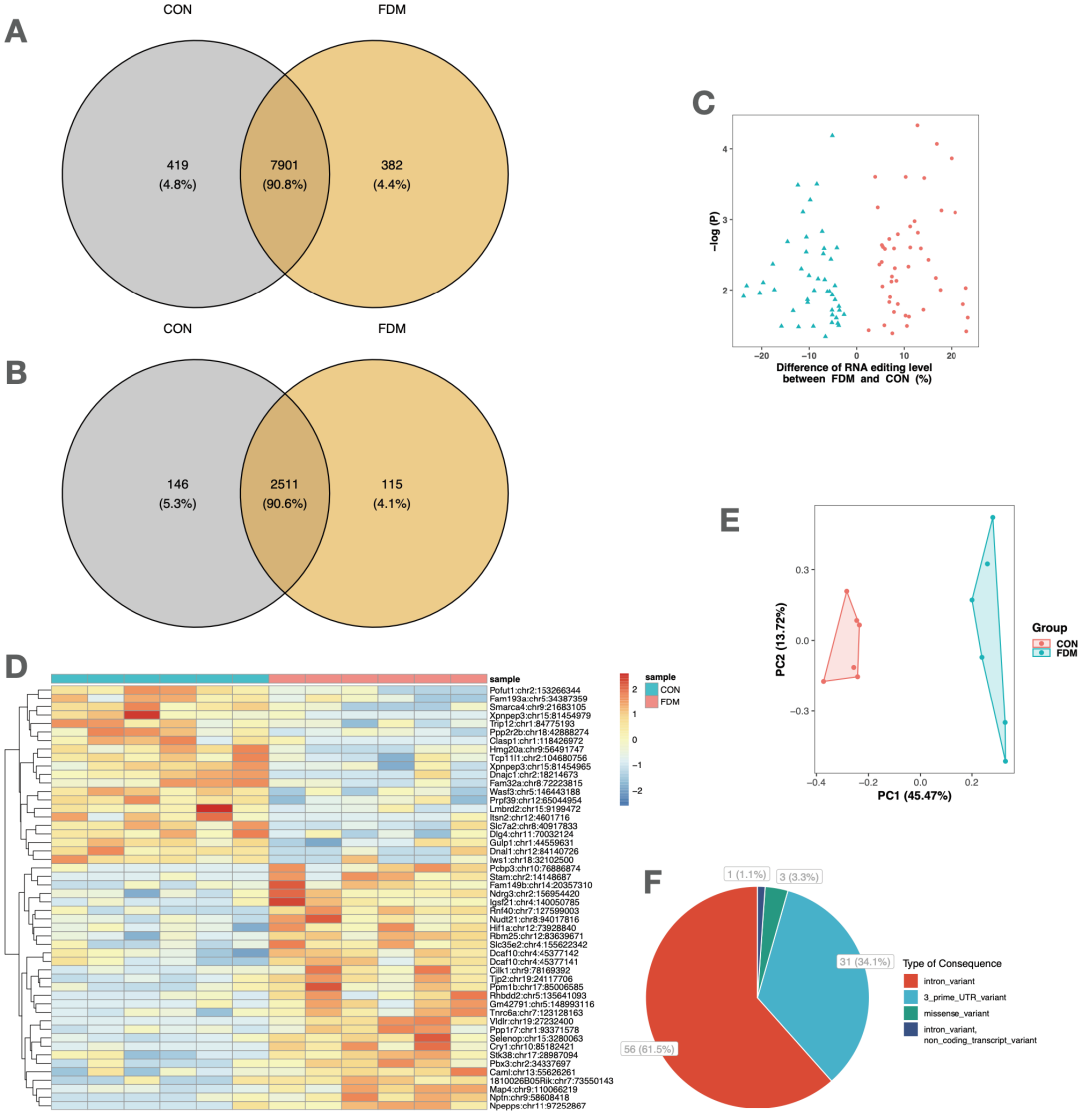
A-to-I RNA editing associated with FDM. Venn plots show the comparison of A-to-I RNA editing sites **(A)** and edited genes **(B)** between FDM and control mice. **(C)** The volcano plot shows the 91 DRE sites between FDM and controls. **(D)** The heatmap shows the editing levels of the top 50 DRE sites in the retina samples. **(E)** Principal component analysis of the 91 DRE sites. **(F)** Functional categories of the 91 DRE sites. FDM, form-deprivation myopia; DRE, differential RNA editing.

The comparison of A-to-I RNA editing sites and genes between FDM and controls is shown in Figure 2. Out of all high-confidence RNA editing sites, 7,901 (90.8%) were common to both groups, while 419 (4.8%) and 382 (4.4%) were unique to controls and FDM, respectively (Figure 2A). Out of 2,772 edited genes, 2,511 (90.6%) were shared by both groups, while 146 (5.3%) and 115 (4.1%) were unique to controls and FDM, respectively (Figure 2B).

A total of 91 A-to-I different RNA editing (DRE) sites in 84 genes were differentially edited between controls and FDM (Figure 2C), with 46 sites in 44 genes upregulated and 45 sites in 42 genes downregulated in FDM. The top 50 DRE sites ranked by *p*-values are shown in Figure 2D. The DRE sites were used to perform PCA, which revealed that the samples formed distinct clusters according to their groups. The first and second principal components accounted for 45.47 and 13.72% of the total variance, respectively (Figure 2E).

Among these DRE sites, three were missense DRE sites, namely, p.Y62C (c.185A > G) in tripartite motif-containing 9 (*Trim9*), p.S316G (c.946A > G) in zinc finger protein 397 (*Zfp397*), and p.S1973G (c.5917A > G) in nucleoporin 214 (*Nup214*). All these missense DRE sites showed decreased RNA editing in FDM.

The expression changes of the edited genes were then evaluated in the mouse retina. Our results found only five of the edited genes showed differential expression between FDM and controls, namely, SUPT6 interacting protein IWS1 (*Iws1*), polymerase (RNA) III (DNA directed) polypeptide H (*Polr3h*), RNA binding motif protein 25 (*Rbm25*), hypoxia-inducible factor 1 alpha subunit (*Hif1a*), and ring finger protein 40 (Rnf40) (Supplementary Table S4).

### Functional enrichments of myopia-associated RNA editing

Our results showed that at least 27 of the 84 differentially edited genes were associated with myopia or related ocular phenotypes in humans or animal models (Table 1). In particular, 16 of them showed a direct link with the myopia phenotype, including tight junction protein 2 (*Tjp2*), solute carrier family 35, member E2 (*Slc35e2*), cryptochrome 1 (*Cry1*), hypoxia-inducible factor 1 alpha subunit (*Hif1a*), poly(rC) binding protein 3 (*Pcbp3*), LMBR1 domain containing 2 (*Lmbrd2*), high mobility group 20A (*Hmg20a*), protein phosphatase 1B magnesium dependent beta isoform (*Ppm1b*), transient receptor potential cation channel subfamily M member 3 (*Trpm3*), CDP-diacylglycerol synthase 2 (*Cds2*), selenoprotein P (*Selenop*), trinucleotide repeat containing 6a (*Tnrc6a*), protein phosphatase 2 regulatory subunit B beta (*Ppp2r2b*), cell adhesion molecule 2 (*Cadm2*), geranylgeranyl diphosphate synthase 1 (*Ggps1*), and prominin 1 (*Prom1*).

**Table 1:** Myopia or related ocular phenotypes in genes with DRE between FDM and control retinas.

No.	Gene symbol	Full name	Myopia or related ocular phenotypes		References
			In humans	In animals	
1	*Vldlr*	Very low-density lipoprotein receptor	NA	Abnormal retinal morphology, choroidal neovascularization, retinal neovascularization, and retina photoreceptor degeneration	Xia et al. (2011)
2	*Rbm25*	RNA binding motif protein 25	NA	Cataract, corneal opacity, and anophthalmia	https://www.mousephenotype.org/data/genes/MGI:1914289
3	*Selenop*	Selenoprotein P	Diabetic retinopathy	Myopia	Youngblood et al. (2019)
4	*Tjp2*	Tight junction protein 2	Myopia	Myopia	Kiefer et al. (2013), Pickrell et al. (2016), and Tedja et al. (2018)
5	*Smarca4*	SWI/SNF related, matrix associated, actin dependent regulator of chromatin, subfamily a, member 4	NA	Coffin-Siris syndrome, microphthalmia, and small-cell carcinoma of the ovary hypercalcaemic type	Errichiello et al. (2017)
6	*Hmg20a*	High mobility group 20A	Axial length	NA	Fuse et al. (2022)
7	*Slc35e2*	Solute carrier family 35, member E2	High myopia	NA	Swierkowska et al. (2021)
8	*Prpf39*	Pre-mRNA processing factor 39	NA	Abnormal lens, retina, and eye morphology	https://www.mousephenotype.org/data/genes/MGI:104602
9	*Cry1*	Cryptochrome 1 (photolyase-like)	NA	Myopia	Stone et al. (2016) and Chakraborty et al. (2018)
10	*Hif1a*	Hypoxia-inducible factor 1, alpha subunit	NA	Myopia	Wu et al. (2018), Zhao et al. (2020), and Ren et al. (2022)
11	*Tnrc6a*	Trinucleotide repeat containing 6a	Keratoconus	NA	Xu et al. (2023)
12	*Ppp2r2b*	Protein phosphatase 2, regulatory subunit B, beta	Corneal astigmatism	NA	Shah and Guggenheim (2018)
13	*Pcbp3*	Poly(rC) binding protein 3	Myopia or refractive error	NA	Tedja et al. (2018) and Currant et al. (2021)
14	*Rhbdd2*	Rhomboid domain containing 2	Retinitis pigmentosa	NA	Ahmedli et al. (2013)
15	*Lmbrd2*	LMBR1 domain containing 2	NA	Myopia	Yang et al. (2021b)
16	*Ppm1b*	Protein phosphatase 1B, magnesium-dependent, beta isoform	NA	Myopia	Li et al. (2022)
17	*Hcn1*	Hyperpolarization-activated, cyclic nucleotide-gated K+ 1	NA	Cone morphological defects in diabetic retinopathy	Han et al. (2022)
18	*Fam135a*	Family with sequence similarity 135, member A	NA	Cataract, abnormal lens, retinal vessel, and vasculature morphology	https://www.mousephenotype.org/data/genes/MGI:1915437
19	*Polg2*	Polymerase (DNA directed), gamma 2, accessory subunit	NA	Retina degeneration	https://www.mousephenotype.org/data/genes/MGI:1354947
20	*Trpm3*	Transient receptor potential cation channel, subfamily M, member 3	Myopia	Microphthalmia; cataract with or without glaucoma, and anterior segment defects; high myopia, astigmatism; attenuated pupillary light response (noxious heat insensitivity)	Hughes et al. (2012), Shiels (2020), Zhou et al. (2021), and Behrendt (2022)
21	*Cds2*	CDP-diacylglycerol synthase (phosphatidate cytidylyltransferase) 2	NA	Myopia	
22	*Sclt1*	Sodium channel and clathrin linker 1	NA	Abnormal eye morphology	https://www.mousephenotype.org/data/genes/MGI:1914411
23	*Cadm2*	Cell adhesion molecule 2	Myopia, glaucoma	Retinoblastoma	
24	*Prom1*	Prominin 1	Retinitis pigmentosa, macular degeneration, cone-rod dystrophy, high myopia, and nystagmus	Retinitis pigmentosa, macular degeneration, cone-rod dystrophy, high myopia, and nystagmus	Yang et al. (2008) and Khan and Bolz (2015)
25	*Ggps1*	Geranylgeranyl diphosphate synthase 1		Myopia, cataract, and abnormal morphology of the lens, vitreous body, and retina in mice	https://www.mousephenotype.org/data/genes/MGI:1354947
26	*Zyg11b*	Zyg-ll family member B, cell cycle regulator	NA	Retinal degeneration	Ma et al. (2022)
27	*Tdrd7*	Tudor domain containing 7	Congenital cataract	Increased eye anterior chamber depth, abnormal lens fiber morphology, cataract, ocular hypertension, ruptured lens capsule, abnormal iris morphology, optic neuropathy, abnormal lens morphology	Barnum et al. (2020)

NA, Not available.

**Figure 3:**
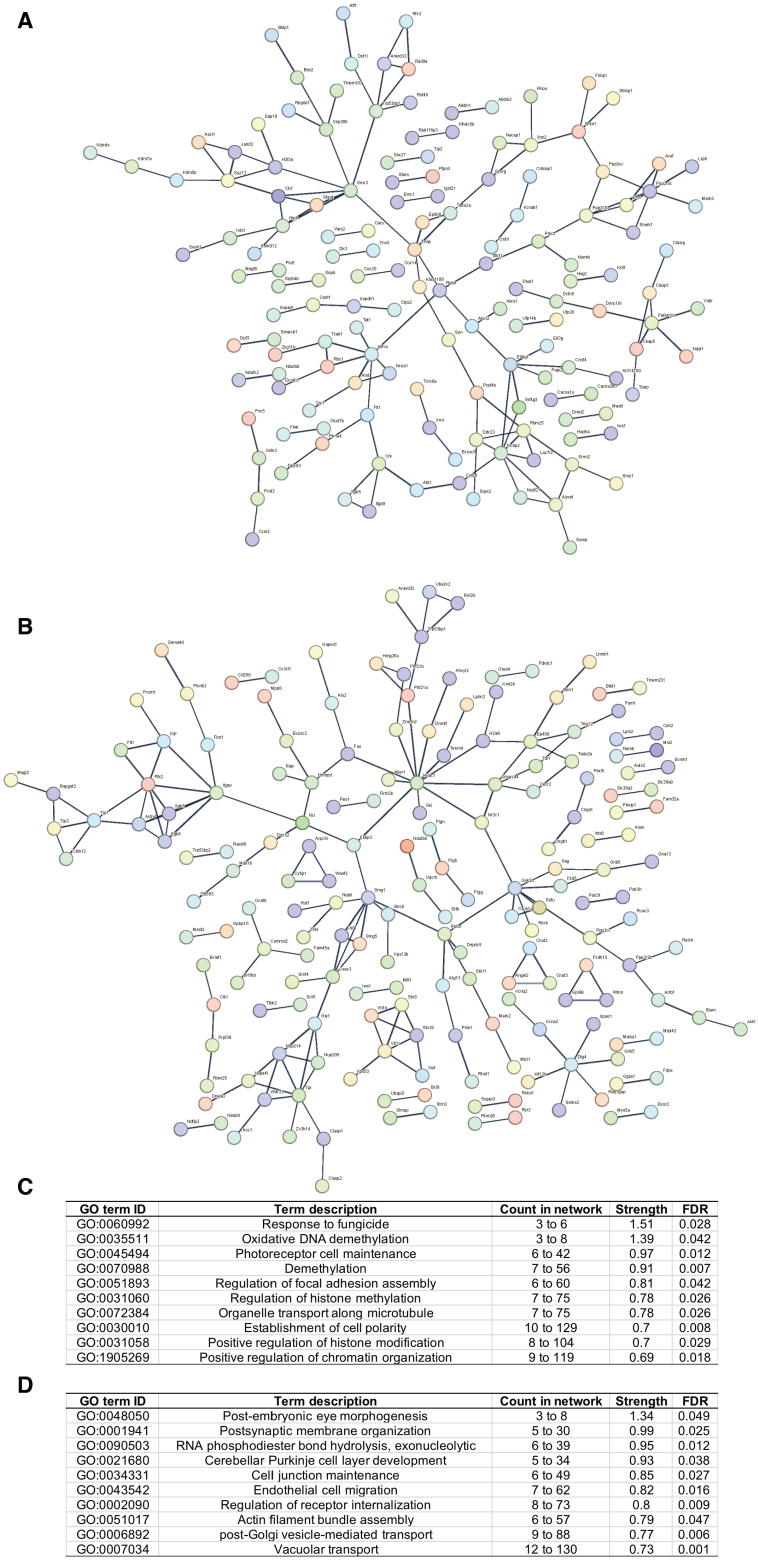
PPI networks and functional enrichment of edited genes enriched in FDM or controls. Networks of edited genes enriched in FDM **(A)** and controls **(B)** are shown. Only interconnected nodes (genes) with high confident correlation (*r* > 0.7) are not shown. The top 10 biological processes related to edited genes enriched in FDM **(C)** and controls **(D)** are listed, respectively.

The PPI networks were constructed, and enrichment analysis was performed for edited genes enriched in a specific group, i.e., with RNA editing sites upregulated or uniquely edited in FDM or controls. The results suggested that edited genes enriched in FDM were mainly involved in biological processes related to response to fungicides, regulation of epigenetics, such as oxidative DNA demethylation, histone methylation, and chromatin organization, and development and structure maintenance, such as photoreceptor cell maintenance, regulation of focal adhesion assembly, organelle transport along microtubules, and establishment of cell polarity. In contrast, edited genes enriched in controls were mainly involved in biological processes related to development and physiological function maintenance, such as post-embryonic eye morphogenesis, exonucleolytic RNA phosphodiester bond hydrolysis, postsynaptic membrane organization, cerebellar Purkinje cell layer development, cell junction maintenance, endothelial cell migration, regulation of receptor internalization, actin filament bundle assembly, and post-Golgi vesicle-mediated transport (Figure 3).

## Discussion

Recent evidence has suggested the substantial role of epigenetics in myopia (Zhou et al., 2012; Seow et al., 2019; Swierkowska et al., 2022). Our current transcriptome-wide study investigated RNA editing changes in the retina of an FDM mouse model and suggested RNA editing as a potentially crucial epigenetic process associated with myopia.

Our analysis revealed dramatic DRE in the mouse retinal transcriptome associated with FDM. Notably, a large proportion of the differentially edited genes had been reported causative or involved with myopia-related phenotypes in humans with existing genetic evidence. *Tjp2* encodes a protein that is a component of the tight junction barrier in epithelial and endothelial cells and is necessary for adequately assembling tight junctions. By using a GWAS approach, SNPs near *TJP2*, *PCBP3, CADM2*, and *TRPM3* were identified to be associated with myopia or refractive errors (Meng et al., 2012; Pickrell et al., 2016; Tedja et al., 2018; Tideman et al., 2021), and *HMG20A* and *PPP2R2B* were associated with axial length and corneal astigmatism, respectively (Shah and Guggenheim, 2018; Fuse et al., 2022). In addition, exome sequencing identified *SLC35E2B* variants in Central European families with high myopia (Swierkowska et al., 2021). Trio-based exome sequencing identified *TNRC6A* variants in keratoconus, a genetic disease causing myopia and astigmatism (Xu et al., 2023). In addition, *PROM1* mutations were reported to cause cone-rod dystrophy with high myopia and nystagmus (Khan and Bolz, 2015).

Apart from those genes with human genetic evidence supporting their role in myopia, other genes with DRE were found to be involved in myopia, ocular development, and related disease phenotypes in animals, such as the development of the retina, retinal vasculature, and the lens, as well as disease phenotypes and processes such as scleral hypoxia, cataract, microphthalmia, or retinal degeneration. Notably, emerging evidence has suggested the role of Hif1a and scleral hypoxia in myopia and refractive errors. *Hif1a* encodes a transcription factor that regulates the cellular response to hypoxia. Scleral hypoxia mediated by Hif1a may contribute to myopia by promoting collagen degradation and extracellular matrix remodeling (Wu et al., 2018; Zhao et al., 2020; Ren et al., 2022). *Vldlr* encodes a receptor for very low-density lipoproteins in lipid metabolism, and *Vldlr* knockout mice show subretinal neovascularization that mimics retinal angiomatous proliferation in human patients (Hu et al., 2008). Such findings were in line with a recent hypothesis that the interaction between excessive myopic eye growth and vascular alterations conferred risks of sight-threatening changes in the disease (Benavente-Perez, 2023). The retina is highly vulnerable to ischemia/hypoxia due to its high oxygen demand and limited vascularity in the inner layers (Yu and Cringle, 2001). In addition, other DRE genes could be involved in the lens and anterior segment development or showed differential expression in myopia or treatment. Cds2 catalyzes the synthesis of phosphatidylserine from phosphatidylcholine, and its expression was reported to be altered in response to a 1% atropine treatment in an FDM guinea pig model (Zhu et al., 2022). Ggps1 is a prenyltransferase with geranylgeranyl diphosphate synthase activity and its expression were changed in chicks between 6 h and 72 h of FDM (Giummarra et al., 2018). Mutations in splicing factor *Prpf39* caused abnormal lens, retina, and eye morphology in mice.^2^ Selenoprotein Selenop was significantly upregulated in the chicken retina after 24 h of imposed defocus treatment (Ohngemach et al., 2004). *TDRD7* is involved in the development of the lens and the anterior segment, and its mutations could cause congenital cataracts in humans (Barnum et al., 2020). The myopia-associated RNA editing in these genes was in line with their essential role in the lens or anterior segment in refraction and development of myopia.

Unexpectedly, our gene enrichment analysis indicated that one of the biological processes related to edited genes enriched in FDM, including those with upregulated or specific RNA editing sites in FDM, was a response to fungicides. In mammalian models, highly selective fungicides, such as MT7 (muscarinic acetylcholine receptor M1 receptor antagonists) and MT3 (muscarinic acetylcholine receptor M4 receptor antagonists), have been reported to prevent experimentally-induced axial myopia (Arumugam and McBrien, 2012). Furthermore, atropine, a muscarinic acetylcholine receptor antagonist, is the only consistently effective medication in slowing myopic progression (Wu et al., 2019). Differentially edited genes involved in response to fungicides included *Hif1a*, steroidogenic acute regulatory protein (*Star*), and lysine demethylase 5B (*Kdm5b*). It was noted that Hif1a could also be involved in response to econazole, which is another fungicide with anti-muscarinic effects (Vass et al., 2018). Several FDM-enriched biological processes were related to epigenetic regulation, such as the regulation of DNA and histone methylation and chromatin organization, indicating the importance of various epigenetic processes and their interplay in myopia. Other FDM-enriched biological processes were related to physiological processes such as photoreceptor cell maintenance. In contrast, biological processes the most enriched in controls emphasized the role of these RNA editing in post-embryonic eye morphogenesis.

The current study demonstrates dramatic changes in RNA editing in the retinal epitranscriptome of an FDM mouse model. Our results were thus limited to the original study’s sample size, and further validation and functional analysis of RNA editing in additional mouse models are therefore needed in future studies.

In conclusion, the current study provided initial evidence pointing toward a potential role played by A-to-I RNA editing during myopia development. Such findings thus warrant further studies on the role of RNA editing in myopia etiology.

## Data availability statement

The original contributions presented in the study are included in the article/Supplementary material, further inquiries can be directed to the corresponding authors.

## Author contributions

X-BP, Y-SH, and H-RP performed the data analysis and wrote the manuscript. Z-YW improved the data analysis pipeline. ZL and Y-YJ participated in the discussion. JW and J-HC conceived the project and planned the experiments. All authors contributed to the final manuscript.

## Funding

This study was supported in part by grants from the National Natural Science Foundation of China (No. 31671311), the “Six Talent Peak” Plan of Jiangsu Province (No. SWYY-127), the Innovative and Entrepreneurial Talents of Jiangsu Province, the Program for High-Level Entrepreneurial and Innovative Talents of Jiangsu Province, the Natural Science Foundation of Guangdong Province/Guangdong Basic and Applied Basic Research Foundation (2019A1515012062), the Taihu Lake Talent Plan, and the Fundamental Research Funds for the Central Universities (JUSRP51712B and JUSRP1901XNC), Wuxi Women and Children’s Health Research Program (FYKY202107).

## Conflict of interest

The authors declare that the research was conducted in the absence of any commercial or financial relationships that could be construed as a potential conflict of interest.

## Publisher’s note

All claims expressed in this article are solely those of the authors and do not necessarily represent those of their affiliated organizations, or those of the publisher, the editors and the reviewers. Any product that may be evaluated in this article, or claim that may be made by its manufacturer, is not guaranteed or endorsed by the publisher.

## Footnotes

^1^ https://www.ebi.ac.uk/ena

^2^ https://www.mousephenotype.org/data/genes/MGI:104602
